# CRISPR/Cas9 from bench to bedside: what clinicians need to know before application?

**DOI:** 10.1186/s40779-020-00292-2

**Published:** 2020-12-08

**Authors:** Zi-Qing Li, Chao-Hong Li

**Affiliations:** 1 Department of Joint Surgery, Shandong Provincial Hospital Affiliated to Shandong First Medical University, Jinan, 250021 China; 2 Department of Basic and Translational Sciences, School of Dental Medicine, University of Pennsylvania, Philadelphia, PA 19104 USA; 3 Department of Histology and Embryology, Zhongshan School of Medicine, Sun Yat-sen University, Guangzhou, 510080 China

**Keywords:** CRISPR/Cas9, Nobel prize, Genome editing, Off-target effect, Ethical concerns

## Abstract

In October 2020, Dr. Emmanuelle Charpentier and Dr. Jennifer Doudna won the Nobel Prize in Chemistry for their pioneering work in precise genome editing using the CRISPR technology. Although CRISPR technology has developed rapidly in the last decade, there are still many uncertainties before eventual use in clinical settings. In this mini review, we summarize the current efforts in addressing the limitations of CRISPR technology and future directions.

Dear editor,

In October 2020, the Nobel Committee announced the award of Nobel Prize in Chemistry to Dr. Emmanuelle Charpentier and Dr. Jennifer Doudna for their pioneering work in precise genome editing with the clustered regularly interspaced short palindromic repeats (CRISPR) technology. The basic features of CRISPR were first recognized by Japanese scientists in 1987 and officially termed as “CRISPR” in 2002, but it was not further developed until the early 2000s, when it was used as a powerful genome editing platform [1, 2]. This technology allows exceptionally precise genome editing in a wide range of species and extends our ability to investigate the contribution of genetic factors to various unexplained phenotypes and diseases. After the rapid development in laboratory settings, CRISPR technology has thunderingly reached the stage of applied biotechnology, and more—gene therapy. Meanwhile, the limitations of this technology, the unknown functions of candidate genes, and the ethical concerns of human use became increasingly emerging before clinical application [3].

In a previous issue of *Military Medical Research*, Prof. Xiao Yang [4, 5] provided an overview of CRISPR/Cas9-mediated genome engineering and its current applications. In the same issue, Dr. Chun-xiao Li and Dr. Hai-li Qian expressed concerns about the limitations of this technology and ethical issues in future use [4, 5]. Indeed, it took only less than 10 years from the development of CRISPR/Cas9 as a basic science research tool to the translation of CRISPR technologies (CRISPR/Cas9-mediated genome editing, CRISPR activation, and CRISPR interference) into powerful therapeutic implement [3, 6]. Uncertainties still exist, and if we do not pay enough attention to evidence-based clinical standards and proceed rushly, there may be consequences that we cannot afford. In this mini-review, we summarize the current efforts in addressing the limitations of CRISPR technology and future directions.

Off-target effects are the most common challenge for all genome editing technologies, and CRISPR/Cas9 is no exception even for its crown of precision and efficiency. Only 22 days after the 2020 Noble Prize was announced, Dr. Dieter Egli’s laboratory published an article entitled “Allele-Specific Chromosome Removal after Cas9 Cleavage in Human Embryos” in *Cell*, emphasizing the significant risk of aneuploidy and other adverse genetic consequences resulting from CRISPR/Cas9 gene editing in early human embryos [7]. This article demonstrated that approximately half of Cas9-induced double-strand breaks (DSB) remained unrepaired after manipulation, followed by chromosomal losses and hemizygous indels after mitosis due to off-target effects in both alleles.

Two core components are required for CRISPR/Cas9 to produce on-target action: 1) a chimeric single guide RNA (sgRNA) that helps Cas9 nuclease to recognize the target DNA sequence; 2) a specific protospacer adjacent motif (PAM) serving as sgRNA recognition site adjacent to the target DNA sequence [4, 8]. Most off-target mutations are due to sgRNA mismatches or recognition by non-specific PAM [8, 9]. A variety of methods, including WGS, GUIDE-seq, Digenome-seq, BLESS, SITE-Seq, CIRCLE-seq, DISCOVER-Seq, GOTI, EndoV-seq, and VIVO, have been developed to detect and evaluate off-target effects [6, 9], and the attempts to solve this off-target issue have never stopped. Currently, engineered Cas9 variants are developed through optimizing guided designs to reduce off-target effects while maintaining editing efficacy, including Cas9-D1135E (improved PAM recognition), Cas9-QQR1 (altered PAM), SpCas9-HF1 (reduced off-target effect), Cas9n/Cas9D10A (single-strand break instead of DSB), xCas9–3.7 (broad PAM specificity), SpCas9-NG (Minimal PAM) and SaCas9-RL (Relaxed PAM) [3, 9]. Also, the cleavage activity of Cas9 nucleases can be attenuated by AcrIIA2 and AcrIIA4 (anti-CRISPR protein) to neutralize the assembled Cas9/sgRNA after the cleavage event [10, 11]. In addition, by using a tissue-specific promoter or chemical inducer, the expression of Cas9 nuclease can be spatially and temporally controlled to avoid DNA cleavage at unintended genomes and to decrease the exposure time of genomes under Cas9 cleavage [12, 13]. These options can be used alone or in combination.

Other limitations of CRISPR technology that will not be elaborated in here include DNA damage-induced toxicity and apoptosis, host immune response to Cas9 and low genome editing efficacy [3, 6, 14], as well as the influence of CRISPR delivery modality on the safety and therapeutic efficacy of target tissues/organs [15].

While celebrating the outstanding achievement of CRISPR technology, we must be aware of ethical controversies and potential risks, as illustrated above and beyond, before clinical applications. Towards this end, the scientific community must strengthen collaboration and communicate with the society at large for further development. New ideas are needed to overcome technical challenges. A set of clearly stated ethical standards must be established to minimize potential harm. Nonetheless, CRISPR technology clearly has vast potential and holds great promises in the fight against human diseases as well as in many other areas with wider impact, such as food shortages and environmental deterioration.

## Data Availability

Not applicable.

## Abbreviations

BLESS: Breaks labeling, enrichment on streptavidin, and next-generation sequencing
Cas9: CRISPR-associated proteins
CIRCLE-seq: Circularization for in vitro reporting of cleavage effects by sequencing
CRISPR: Clustered regularly interspaced short palindromic repeats
DSB: Double-strand breaks
EndoV-seq: Endonuclease v sequencing
GOTI: Genome-wide off-target analysis by two-cell embryo injection
GUIDE-seq: Genome-wide, unbiased identification of DSBs enabled by sequencing
PAM: Protospacer adjacent motif
SITE-Seq: Selective enrichment and identification of adapter-tagged DNA ends by sequencing
sgRNA: single guide RNA
VIVO: Verification of in vivo off-targets
WGS: Whole-genome sequencing

## References

[1] Lander ES (2016). The heroes of CRISPR. Cell..

[2] Dort EN, Tanguay P, Hamelin RC (2020). CRISPR/Cas9 gene editing: an unexplored frontier for forest pathology. Front Plant Sci.

[3] Uddin F, Rudin CM, Sen T (2020). CRISPR gene therapy: applications, limitations, and implications for the future. Front Oncol.

[4] Yang X (2015). Applications of CRISPR-Cas9 mediated genome engineering. Mil Med Res.

[5] Li CX, Qian HL (2015). A double-edged sword: CRISPR-Cas9 is emerging as a revolutionary technique for genome editing. Mil Med Res..

[6] Hsu MN, Chang YH, Truong VA, Lai PL, Nguyen TKN, Hu YC (2019). CRISPR technologies for stem cell engineering and regenerative medicine. Biotechnol Adv.

[7] Zuccaro MV, Xu J, Mitchell C, Marin D, Zimmerman R, Rana B, et al. Allele-specific chromosome removal after Cas9 cleavage in human embryos. Cell. 2020. 10.1016/j.cell.2020.10.025. 10.1016/j.cell.2020.10.025 33125898

[8] Kleinstiver BP, Prew MS, Tsai SQ, Topkar VV, Nguyen NT, Zheng Z (2015). Engineered CRISPR-Cas9 nucleases with altered PAM specificities. Nature..

[9] Manghwar H, Li B, Ding X, Hussain A, Lindsey K, Zhang X (2020). CRISPR/Cas systems in genome editing: methodologies and tools for sgRNA design, off-target evaluation, and strategies to mitigate off-target effects. Adv Sci (Weinh).

[10] Liu L, Yin M, Wang M, Wang Y (2019). Phage AcrIIA2 DNA mimicry: Structural basis of the CRISPR and anti-CRISPR arms race. Mol Cell.

[11] Shin J, Jiang F, Liu JJ, Bray NL, Rauch BJ, Baik SH (2017). Disabling Cas9 by an anti-CRISPR DNA mimic. Sci Adv.

[12] Singh K, Evens H, Nair N, Rincon MY, Sarcar S, Samara-Kuko E (2018). Efficient in vivo liver-directed gene editing using CRISPR/Cas9. Mol Ther.

[13] Lu J, Zhao C, Zhao Y, Zhang J, Zhang Y, Chen L (2018). Multimode drug inducible CRISPR/Cas9 devices for transcriptional activation and genome editing. Nucleic Acids Res.

[14] Bourgeois JS, Smith CM, Ko DC. These are the genes you're looking for: finding host resistance genes. Trends Microbiol. 2020. 10.1016/j.tim.2020.09.006. 10.1016/j.tim.2020.09.006 PMC7969353 33004258

[15] Xu X, Wan T, Xin H, Li D, Pan H, Wu J (2019). Delivery of CRISPR/Cas9 for therapeutic genome editing. J Gene Med.

